# Supplementary material for: Behavioral signatures of post-decisional attention in preferential choice
Source: eLife. 2026 Jul 28;15:RP110729. doi: 10.7554/eLife.110729 (PMC13412323; doi:10.7554/eLife.110729)
Supplement: Peer review history

# Editor report: eLife Assessment

**DOI:** 10.7554/eLife.110729.3.sa0

**Authors:** Emilio Salinas

**Affiliations:** Wake Forest University School of Medicine United States

**Keywords:** Convincing, Important

This study uses **convincing** modeling methods and analyses of rich behavioral datasets to investigate the role of attention in value-based decision-making; for instance, as when choosing between two snacks. The results are **important**, as they challenge existing theories that assume that paying attention to an available option biases the eventual choice toward that option. The results suggest that the correlation between attention and decision-making is formed largely after rather than before the (internal) choice process has terminated, a finding that offers an intuitively appealing rethinking of how attention and decision-making processes interact during value-based choices.

# Referee report: Reviewer #1 (Public review)

**DOI:** 10.7554/eLife.110729.3.sa1

[Editors' note: this version has been assessed by the Reviewing Editor without further input from the original reviewers. The authors have addressed the weaknesses raised in the previous round of review.]

Summary:

This study examines whether gaze direction actively shapes choice during food preference decisions or whether gaze and choice evolve largely independently until the moment of commitment. The established framework in this context, the aDDM, assumes that gaze causally biases the accumulation of evidence in favour of the fixated item. The authors show convincingly that this model fails to fit key behavioural patterns across several datasets, as do other published models that make the same assumption. The authors propose an alternative model (Post-Decision-Gaze or PDG) in which gaze and decision formation are decoupled: gaze does not influence the decision process, nor is it drawn toward the ultimately chosen item, until after the decision threshold is reached. Only during the motor execution period (after commitment) is gaze directed to the chosen option. They demonstrate that this model fits several observed patterns better than the aDDM and related variants.

Strengths:

The work thoroughly considers multiple models and datasets. It advances an interesting alternative perspective on gaze-decision interactions and highlights meaningful shortcomings in existing models. The authors take the time to explain how modelling assumptions produce specific patterns in the data, which is certainly insightful to readers interested in the modelling of value-based decision making.

Weaknesses:

It is unclear to what extent the model's success relies on the way non-decision time is formalised in the model. In the proposed PDG model, non-decision time is decomposed into separate visual encoding, saccadic execution, and manual execution components. Several values (assumed or recovered) do not match known physiological or behavioural ranges. This is a common issue in the literature, and the authors may want to address it in light of broader work discussing what non-decision time consists of in both manual and saccadic actions (e.g., Bompas et al., 2024, Non decision time: the Higgs boson of decision, Psychological Review).

# Referee report: Reviewer #2 (Public review)

**DOI:** 10.7554/eLife.110729.3.sa2

Summary:

Zylberberg et al. reanalyze eye-tracking and behavioral data to test two predictions of the attentional Drift Diffusion Model, finding that these predictions are not met. Similarly, predictions of normative models (inspired by rational inattention) are not in line with the data, and the authors propose a post-choice model of attention. This model better accounts for the two effects but also does not account for all patterns, so the authors conclude that eye movements most likely reflect both pre- and post-decisional processes.

Strengths:

A clear strength is the systematic falsification-based approach of the paper, establishing (partially) new predictions and testing to what extent these are met by extant models and by a newly developed theory. The authors do a good job in providing intuitions behind the effects and the reasons why models such as the aDDM predict them. The paper is of substantial relevance for the field, as it shows that effects pertaining to the last fixation(s) should be interpreted with caution. Another strength is the paper's transparency as the authors clearly acknowledge that their new model does not do a perfect job either.

Weaknesses:

The paper focuses on analyzing the Krajbich 2010 data, but shows that the second effect replicates in many other datasets. A more principled approach, in which both effects are analyzed and presented for all datasets, would be more convincing. The results should then be shown together for clarity/readability.

Similarly, it would be nice to show to what extent the models' predictions depend (not depend) on using the best-fitting parameter values (are there any parameter settings under which the two effects are not predicted?)

# Referee report: Reviewer #3 (Public review)

**DOI:** 10.7554/eLife.110729.3.sa3

Summary:

In this study, the authors reanalyzed choice, RT and gaze datasets collected from human subjects performing a food-choice task. They show that models that posit a causal role for attention in shaping the decision-making process fail to account for empirical observations in the data. These include the attentional drift diffusion model (aDDM) and models that derive attention-choice associations from an optimal policy. The authors show that a model that assumes that gazes are directed towards the chosen option after decision commitment captures more (but not all) empirical findings, suggesting that attention may reflect decisions once they are made instead of contributing to their formation. However, this post-decision-gaze (PDG) model failed to capture all aspects of the data, suggesting that gaze may reflect both decisional and post-decisional operations, and existing models are still missing some features of the gaze-directing process. The authors provide convincing evidence that post-decision gaze explains a number of empirical findings in this task.

Strengths:

(1) The analyses are generally appropriate, and the conclusions are supported by the data.

(2) The study was rigorous, as the authors considered a number of alternative possible models for behavior, and evaluated their performance based on a wide range of qualitative predictions (as opposed to exclusively relying on model comparison).

(3) The proposal that gaze may largely reflect post-decisional processes is interesting, and as far as I am aware, novel.

Weaknesses:

There was limited discussion about why one might allocate attention post-decision. I would have appreciated more discussion on the potential functional consequences or implications of post-decision gaze.

# Author comment: Author response

**DOI:** 10.7554/eLife.110729.3.sa4

**Authors:** Ariel Zylberberg, Ian Krajbich, Michael N Shadlen

**Affiliations:** Columbia University New York United States; University of California, Los Angeles Los Angeles United States; Columbia University New York United States

The following is the authors’ response to the original reviews.

**Public Reviews:**

**Reviewer #1 (Public review):**

It is unclear to what extent the model's success relies on the way non-decision time is formalised in the model. In the proposed PDG model, non-decision time is decomposed into separate visual encoding, saccadic execution, and manual execution components. Several values (assumed or recovered) do not match known physiological or behavioural ranges. This is a common issue in the literature, and the authors may want to address it in light of broader work discussing what non-decision time consists of in both manual and saccadic actions (e.g., Bompas et al., 2024, Non decision time: the Higgs boson of decision, Psychological Review).

In particular, the "saccadic execution" parameter appears far too long and too variable to reflect merely execution; instead, it likely includes decisional components. This would make more sense since manual and saccadic planning essentially rely on distinct brain areas, hence it seems unrealistic that crossing a single threshold would trigger both manual and saccadic execution. Similarly, recovered manual non-decision times are substantially longer (though not more variable) than expected motor execution durations for button presses. These patterns suggest that parts of what the model treats as non-decision time are likely decisional in nature, although perhaps related to "action decision" rather than the "value-based decision" of interest to the authors. To what extent these two processes neatly follow each other or overlap could be usefully considered.

We have added a paragraph to the Discussion explaining how our model’s estimates of sensory and motor latencies relate to corresponding values inferred from physiology or behavioral manipulations (e.g., Bompas et al., 2024). Specifically, we write:

“The key assumption of the *PDG* model is that there is a delay between the moment a choice is internally committed and the moment it is externally reported with a key press. Because eye movements are typically faster than manual responses (𝜏_e_ < 𝜏_m_ in our simulations), this delay creates a window during which gaze can already be directed toward the covertly chosen item before the response is formally registered. We do not interpret these non-decision latencies as irreducible physiological minima for moving the eyes or pressing a button (Bompas et al., 2025). Rather, they are inferred indirectly by fitting an additive non-decision-time parameter to the behavioral data, which we decompose into a sensory delay (𝜏_s_) and a manual execution delay (𝜏_m_). Values of 𝜏_e_ are then chosen so that the model reproduces the observed magnitude of the behavioral effects. This estimation procedure has important limitations. Some participants show relatively “flat” chronometric functions: response times vary little with value despite otherwise normal psychometric performance. Such patterns likely reflect processes not explicitly represented in the model, including procrastination, reduced motivation, task-unrelated thought, or noise in item ratings. Within a drift-diffusion framework, however, these cases are accommodated by assigning a long non-decision time together with a short evidence-accumulation period (Table S1). Consequently, some estimated non-decision times are substantially longer than would be expected if they represented only sensory and motor delays. A further limitation is conceptual. We model non-decision time as occurring either before or after evidence accumulation, whereas in reality decisional and non-decisional components are likely temporally interleaved (Graziano et al., 2011). This simplification may also inflate the recovered latency estimates. With these caveats in mind, sensory and oculomotor delays on the order of 300 ms remain broadly plausible, although they likely lie near the upper end of a realistic range. The estimated eye-movement latency is especially long. For instance, in monkeys trained to report simple perceptual decisions with a saccade, roughly 100 ms elapses between the threshold-crossing signal in parietal cortex (or the superior colliculus) and the executed eye movement (Roitman and Shadlen, 2002; Stine et al., 2023). Crucially, however, varying the assumed non-decision latencies across a reasonable range does not alter the qualitative predictions of the model (Fig. 8).”

Further, we have added a parameter sensitivity analysis. Importantly, although the magnitude of the predicted effects depend on the non-decision latencies, the qualitative aspect of these predictions do not (new Figure 8). Specifically, (i) the increasing tendency to look at the ultimately chosen item as time elapses (new Fig. 8A), (ii) the lack of an interaction between the last-fixation bias and overall value (Fig. 8B), and (iii) the absence of an effect of choice consistency on Δdwell (Fig. 8C) are all findings that are independent of 𝜏_e_.

**Reviewer #2 (Public review):**

The paper focuses on analyzing the Krajbich 2010 data, but shows that the second effect replicates in many other datasets. A more principled approach, in which both effects are analyzed and presented for all datasets, would be more convincing. The results should then be shown together for clarity/readability.

Following this suggestion (and the reviewer’s elaboration below), we have substantially restructured the manuscript. Both *aDDM* predictions are now presented together (new Fig. 2), and Figs. 3–4 test these predictions across multiple food-choice datasets. In doing so, we no longer treat the data from Krajbich et al. (2010) separately, and we extend the analysis of the last-fixation–choice association (MELFB) to additional datasets. We note that the same datasets could not be used in both Figs. 3 and 4, as some lack information on the final fixation required for the MELFB analysis. Nevertheless, results are highly consistent across datasets and align with findings from a recent study by Ting & Gluth (2025), which independently identified and examined one of our key predictions; this work is now cited in the revised manuscript. Finally, to reduce redundancy, we have consolidated all aDDM variants and optimal models into a single figure (new Fig. 10).

Similarly, it would be nice to show to what extent the models' predictions depend (not depend) on using the best-fitting parameter values (are there any parameter settings under which the two effects are not predicted?)

The key predictions of the model depend on the difference between the manual (𝜏_m_) and eye-movement-related (𝜏_e_) latencies. We have now added a parameter-sensitivity analysis to show how the model predictions depend on this difference. The new analysis shows that while the quantitative predictions do depend on the precise latency values, the results are qualitatively similar across values of 𝜏_e_ (new Figure 8).

**Reviewer #3 (Public review):**

There was limited discussion about why one might allocate attention post-decision. I would have appreciated more discussion on the potential functional consequences or implications of post-decision gaze.

Thank you for this suggestion. We added a new paragraph to the discussion (paragraph #2), where we argue that it is sensible for a decision maker to direct the gaze to the chosen item once a covert choice commitment has been made, as the benefits of attending to a stimulus do not end with the decision itself. Specifically we now write:

“Instead, these observations are better explained by a post-decision account of the gaze-choice association that is, one in which gaze shifts to the selected item after a covert commitment to a choice. We argue that directing gaze to the chosen item after a covert choice commitment is sensible, as the benefits of attending to a stimulus do not end with the decision itself. In naturalistic settings, for instance, selecting a food item is typically followed by the action of reaching toward it, where visual attention supports spatial localization and motor planning for the upcoming action. Although participants in our computerized task did not physically act on their choices, these sensorimotor processes are likely highly automatized and may still be engaged by default, even when not strictly required. Beyond motor preparation, post-decisional attention may also serve additional functions, such as facilitating sensory anticipation of the reward, supporting metacognitive evaluation of the decision, and contributing to value updating for future choices. From this perspective, a degree of attentional “stickiness” whereby the chosen item remains preferentially attended after commitment could emerge as an effectively optimal policy once these post-decisional processes are taken into account. Moreover, a specific feature of the task design may further reinforce this tendency: in the snacks paradigm, the unchosen item typically disappears from the screen immediately after a response is registered. It is therefore plausible that directing gaze to the chosen item after commitment partly reflects anticipation of the imminent disappearance of the unchosen option. To disentangle these mechanisms, it would be interesting for future work to test whether this attentional bias persists when the chosen item, rather than the unchosen one, is the stimulus that disappears upon response.”

**Recommendations for the authors:**

**Reviewer #1 (Recommendations for the authors):**

Major Comments:

(1) Framing of the modelling approach

The manuscript would benefit from acknowledging the known limitations of DDM-based frameworks, especially given that the entire study is conducted within these constraints. The introduction highlights successes of the DDM, but the manuscript does not mention any of its conceptual or empirical limitations.

We are unsure about what specific limitations the reviewer has in mind, but we have added a paragraph to discussion mentioning some limitations, like the inflation of the non-decision times and the difficulty of interpreting the fit parameters (Paragraph #5 of Discussion: “The key assumption of the PDG model is that there is...”).

(2) Dependence on non-decision time assumptions

The alternative model's explanatory power appears to rely heavily on assumptions regarding the decomposition of non-decision time: fixed visual encoding (𝜏_s_ = 0.3 s), manual non-decision time (𝜏_m_; two free parameters), and saccadic execution (𝜏_e_; fixed parameters μ_e_ = 0.35, σ_e_ = 0.11).

- 𝜏_e_ is substantially longer and more variable than typical saccadic execution times, suggesting it likely incorporates decisional components.

- Estimated 𝜏_m_ values are approximately twice as long as known manual execution durations.

- σnd is more plausible, implying that variability is captured correctly but mean durations are not.

Together, these points raise the possibility that portions of what the model treats as non-decision time are in fact part of a (action) decision process. Only then does it make sense to assume that Tm is usually larger than Te. If Tm and Te were truly execution delays, then Tm would always be larger than Te.

You may find it helpful to consider the framework in Bompas et al. Psych Review (2024), which discusses in detail what non-decision time is likely to comprise across effectors.

Thank you we have added (i) a sensitivity analysis showing that our results are robust to changes in the specific value used for the eye movement related latencies (new Fig. 8), and (ii) a new paragraph in Discussion addressing the issue of the mismatch between our parameter estimates and the manual and saccadic execution times (Paragraph #5 of Discussion: “The key assumption of the PDG model is that there is...”).

(3) Code availability.

The authors should consider sharing all relevant code and data publicly.

We agree, we now share the code and data on GitHub and indicate so in the revised manuscript.

Minor Comments:

(1) Lines 74-77. These are not worded as predictions but as questions; one tests predictions, but answers questions. I feel it would be clearer to stick to predictions (like in the abstract), and the introduction could benefit from explaining these predictions in a bit more detail (I found it difficult to get my head around these predictions from the intro text only).

We rewrote the section in the introduction where we provide a gist of the model predictions (last paragraph of Introduction). We agree with the reviewer that the previous explanation was not clear.

(2) It is confusing that panel B appears to the left of panel A in Figure 2.

We agree. We have restructured the manuscript (following the suggestion of another reviewer), and now Figure 2 has changed and the panels follow a more logical order.

(3) Figure 3C - remove MATLAB toggles.

Yes, thanks.

(4) Figure 5A shows the proportion of left choices, but the text and legend refer to right choices.

Good catch, thank you.

**Reviewer #2 (Recommendations for the authors):**

This may appear self-serving, but the authors seem to be unaware of some highly relevant work from our group. Most importantly, in a recent publication (Ting & Gluth, 2024, JEP General), we have already looked at the dependency of the last- (or final-) fixation bias on overall value in value-based (VB) and perceptual (P) decisions. In VB, we found a negative effect; in P we did not find a significant effect. This is largely consistent with the current results, showing a negative but not significant trend. Another relevant work is Gluth et al. (2020, Nat Hum Behav), where we extended the aDDM by assuming that the probability to fixate on an option is a function of the accumulated evidence for that option. It would be interesting to know whether this assumption changes the predictions of the aDDM. Finally, we just published a new theory on how people search for information to make efficient value-based decisions (Gluth et al., in press, Psychol Rev; https://osf.io/preprints/psyarxiv/3qzak_v2). Although this theory focuses on multi-attribute choices, it can be applied to "simple" choices, too (by assuming that there is only one attribute = value). Interestingly, while the model also mispredicts a (slight) increase of the last-fixation bias with overall value, it correctly predicts the independency of the dwell-time advantage effect on choice consistency as well as the small increase of the effect with RT (attached here is a figure to show this: [https://elife-rp.msubmit.net/elife-rp_files/2026/01/22/00149589/00/149589_0_attach_9_477122. pdf], and the match with the empirical data shown in Figure 3B and 12 is striking). In general, the model shares many features of the Callaway and Jang models, but does not need to assume a biased value prior, which the authors suggest is responsible for the misprediction of the second effect. I leave it up to the authors to discuss this new theory, but I wanted to point this out.

Thank you for pointing this out; these are all relevant points and studies.

We now note that the first of our predictions has recently been identified and tested by Ting and Gluth (2025).

We also considered extending the manuscript with a variant of the model proposed by Gluth et al. (Psychological Review, 2026). In fact, we attempted to fit this model to the Krajbich et al. (2010) dataset under the assumption that the duration of each sampling epoch is a free parameter. We find this model very interesting. However, in our current implementation it appears to make the same qualitative prediction as the aDDM, namely that ΔDwell depends on choice consistency (see Author response image 1).

Given this, we have decided not to include these results in the manuscript. It remains possible that with further development particularly with a more realistic specification of fixation durations (e.g., allowing them to depend on value) the model could account for the full set of observed effects. We think this would be best addressed in a separate study.

That said, we do find the model promising, as it provides a better account than most of the alternative models we explored for the patterns shown in panels D, H, and I.

**Author response image 1.** Fits of a variant of the MACS model (Gluth et al. 2026) to the data of Krajbich et al. (2010).

The paper would benefit substantially from restructuring. The aDDM's predictions are provided first, together with the empirical data, and then the optimal models are discussed. But Figure 2 shows all of this together. Later, the new (PDG) model is elaborated, and its predictions are shown. Towards the end of the results, variations of the aDDM and combinations of aDDM and PDG are shown in a series of figures (8-11), followed by a last figure showing one of the tested effects in other datasets. All of this feels pretty much thrown together without a clear structure. For instance, the aDDM and the optimal models could be described together (or the optimal models get a separate figure). The additive variants could be described earlier. And some figures could be put into the supplement. And the empirical results of the different studies could be shown together.

We fully agree with this suggestion. We have now restructured the manuscript along the lines proposed by the reviewer (see the more detailed explanation of the restructuring in our response to the public comments).

I strongly suggest avoiding the term "influence" in the y-axis of Figure 2, upper row, as it implies causality. Similarly, in line 182, the term "causal influence" is used in the context of the Callaway model, but as far as I know, this is not what the model assumes.

We replaced the y-axis label with “Association of last dwell with choice (β)”

**Reviewer #3 (Recommendations for the authors):**

(1) Figure 2 - Panel labels for A and B are reversed?

We have restructured the manuscript (following the suggestion of another reviewer), and now Figure 2 has changed.

(2) Does 3C include a .pdf screenshot?

Thank you, it’s a Matlab bug on Mac. I guess they want us to switch to Python -:)

(3) Figure 4 - It would be helpful if the green line were defined in the figure legend.

Added

(4) The effect size in 5B looks much more dramatic than in 2B(A?) - Is this for one example subject as opposed to all subjects? Please clarify what is different about the data.

We are no longer showing the psychometric functions in Figure 2.

(5) Line 252 - they say they compared the probability of choosing the right item (Fig. 5B) by the y-labels of that figure, which are all p(choose left).

Yes, corrected now.

(6) In general, they reference the subpanels of Figure 5 out of order, which causes the reader to jump around. They might consider reordering the panels of the figure so they follow the ordering of descriptions in the text.

We agree, we have rearranged the figure panels to follow the ordering of the descriptions in the text.
